# A Man with an Umbilicated Papule of the Hand: What Is Your Diagnosis?

**DOI:** 10.1155/2010/524021

**Published:** 2010-05-31

**Authors:** Deba P. Sarma, Meredith Cox, Paige Walter, William Crisler, Christopher Huerter

**Affiliations:** ^1^Department of Pathology, Creighton University Medical Center, Omaha, NE 68131, USA; ^2^Department of Dermatology, Creighton University Medical Center, Omaha, NE 68131, USA

## Abstract

*Introduction*. Ecthyma contagiosum is a zoonotic disease caused by the parapoxvirus that causes “sore mouth” in sheep and goats and orf in human. *Case Presentation*. A 61-year-old sheep farmer presented with a painful non-pruritic lesion on the left hand that had been present for approximately 5 weeks. Physical examination demonstrated a 1 cm pearly, umbilicated papule with raised borders. A biopsy showed an asymmetrical nodule with parakeratotic crust and acanthosis with thin epidermal strands extending deeply in the underlying dermis. Marked edema, capillary proliferation and extensive lymphocytic infiltration was also present. One red intranuclear inclusion was identified in an epidermal keratinocyte. A diagnosis of human orf (ecthyma contagiosum) was made. *Conclusion*. Infected sheep and freshly vaccinated sheep or goats are the reservoir for human infection. After an incubation period of 3–7 days, parapoxvirus infections produce 1–3 painful lesions measuring 1-2 cm in diameter. The natural history of the disease is complete resolution and no treatment is indicated. Prevention of echthyma contagiosum in ruminants through vaccination is thought to be the best way to control infection.

## 1. Case Synopsis

**Figure 1:**
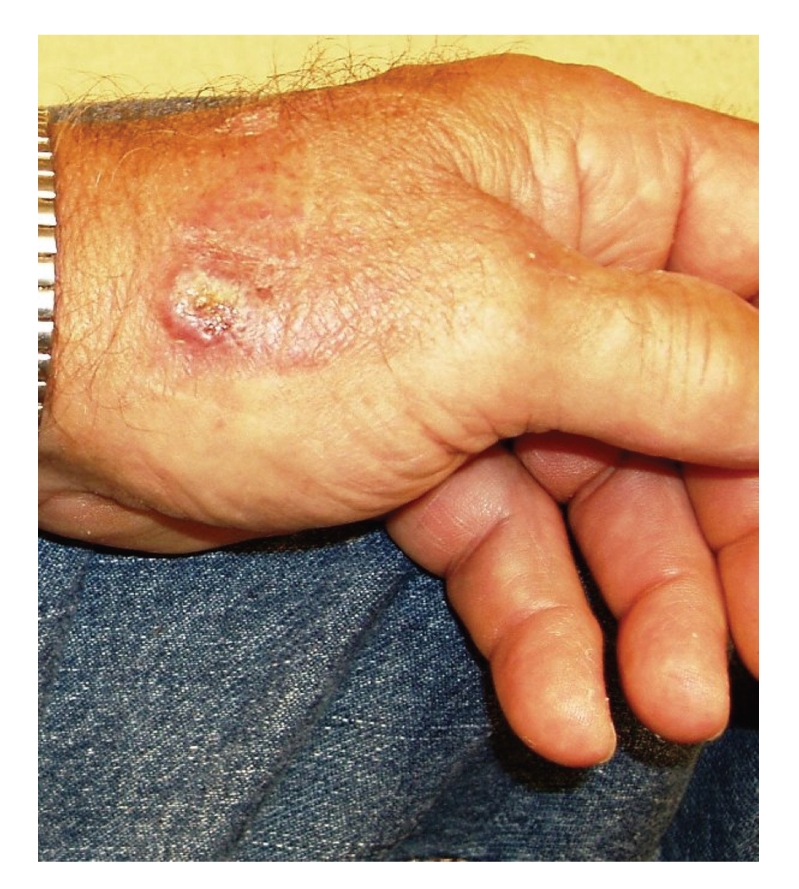
Clinical picture.

A 61-year-old sheep farmer presented with a painful nonpruritic on the left hand that lesion had been present for approximately 5 weeks. He neither had previous history of similar lesions nor did he have a history of cancer or other dermatologic conditions. Physical examination demonstrated a 1 cm pearly, umbilicated papule with raised borders (Figure 1).

**Figure 2:**
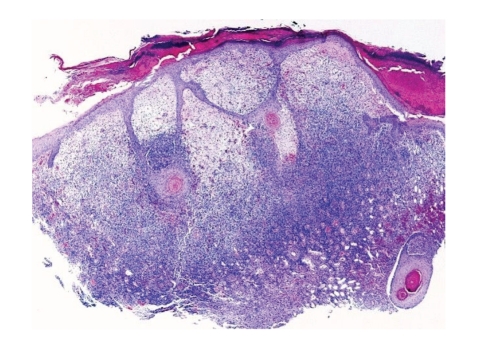
Microscopic appearance: raised papule with parakeratotic crust and acanthosis with thin epidermal strands extending deeply in the underlying dermis. The upper dermis is markedly edematous with marked capillary proliferation. The deeper dermal part of the lesion is composed of reactive lymphoid infiltrates.

**Figure 3:**
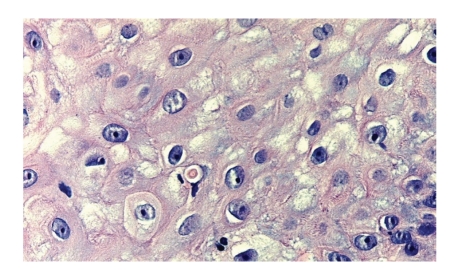
One red intranuclear inclusion in the keratinocyte of the epidermis.

A biopsy (Figure 2) showed an asymmetrical nodule with parakeratotic crust and acanthosis with thin epidermal strands extending deeply in the underlying dermis. The upper dermis showed marked edema and capillary proliferation. The deeper dermal part of the lesion showed extensive lymphocytic infiltration. One red intranuclear inclusion was identified in an epidermal keratinocyte (Figure 3).

## 2. What Is Your Diagnosis?

### 2.1. Diagnosis: Human Orf (Ecthyma Contagiosum)

**Case Synopsis**

A 61-year-old sheep farmer presented with a painful nonpruritic ulcerated lesion on the left hand that had been present for approximately 5 weeks. He neither had previous history of similar lesions nor did he have a history of cancer or other dermatologic conditions. Physical examination demonstrated a 1 cm pearly, umbilicated papule with raised borders (Figure 1).

A biopsy (Figure 2) showed an asymmetrical nodule with parakeratotic crust and acanthosis with thin epidermal strands extending deeply in the underlying dermis. The upper dermis showed marked edema and capillary proliferation. The deeper dermal part of the lesion showed extensive lymphocytic infiltration. One red intranuclear inclusion was identified in the epidermal keratinocyte (Figure 3).

## 3. Discussion

Our patient was a sheep farmer who presented with a raised umbilicated pearly lesion of his left hand that was ulcerated with raised borders. Clinically, it could easily be mistaken for a basal cell carcinoma or squamous cell carcinoma. The central umbilication could also suggest keratoacanthoma or a lesion caused by molluscum infection though such lesions are usually much smaller. Milker's nodules should be considered too. Both the clinical history of sheep farming and the microscopic features including the eosinophilic intranuclear inclusion body in the keratinocyte suggest a diagnosis of human orf (ecthyma contagiosum). The diagnosis may be further confirmed by electron microscopy done on the fluid obtained from the orf lesion showing ovoid cross-hatched virions [1]. Polymerase chain reaction (PCR), although not readily available, can definitely identify orf virus from frozen tissue specimens, vesicle material, or scab debris from orf lesions [2].

Ecthyma contagiosum is a zoonotic disease caused by the parapoxvirus that causes “sore mouth” in sheep and goats and orf in human. In ruminants, it is evidenced by exudative lesions found on the muzzle, eyelids, oral cavity, feet, or external genitalia. It is more common in younger animals. The disease in ruminants is highly contagious to humans and other animals; infected sheep and also freshly vaccinated sheep or goats are the source of infection to people. Transmission can be by direct contact with lesions or indirectly from contaminated object such as hair or clothing [3].

Parapoxvirus is made up of a dense DNA core surrounded by a less dense capsid and 2 narrow electron dense outer layers. After an incubation period of 3–7 days, parapoxvirus infections produce 1–3 painful lesions measuring 1-2 cm in diameter. During the next 6–8 weeks, the lesion passes through 6 clinical stages: maculopapular, target, acute weeping, nodular, papillomatous, and finally regressive stages [4].

Microscopically, in the maculopapular stage, there is vacuolization of cells in the upper third of the stratum malpighii leading to multilocular vesicles. Eosinophilic intranuclear or cytoplasmic inclusion bodies can be seen. Vacuolated epidermal cells with inclusion bodies characterize the target stage. Ballooning degeneration also occurs in the target stage and affects keratinocytes rupture with a tendency to coalesce and produce reticulated vesicles. Additionally, in the epidermis, there is an elongation of the rete ridges. Many newly formed dilated capillaries and a mononuclear infiltrate are present in the dermis. This is followed by the acute weeping stage which is characterized by necrosis and a massive infiltrate of mononuclear cells throughout the dermis. Some biopsies of orf may have a marked reactive lymphoid infiltrate with CD30 positive T cells mimicking lymphoma. On progression to the nodular stage, a lichenoid reaction with a high percentage of histiocytes is seen in the skin. In the final papillomatous stage finger-like downward projections are displayed in the epidermis along with vasodilatation and chronic inflammation in the dermis. This results in resolution and regression of the lesion [5]. 

The natural history of the disease is complete resolution and no treatment is indicated. But antiseptic agents to prevent the superinfection and in some selected cases imiquimod can be applied [6]. Although, immunity is short lived, reinfection frequently appears but no human-to-human transmission occurs. Investigation into prevention of ecthyma contagiosum in ruminants through vaccination is thought to be the best way to control infection. Vaccines are available that offer some efficacy in sheep but do not prevent disease in goats. Research into effective and economical vaccines is ongoing. If infection is controlled in the ruminant population, human infection and its economic and environmental consequences should decrease [5, 7].
